# Which Phenotypes Should We Include in the Hypoplastic Left Heart Syndrome?

**DOI:** 10.1177/21501351231181313

**Published:** 2023-09-22

**Authors:** Robert H. Anderson, Diane E. Spicer, Adrian Crucean

**Affiliations:** 1 Biosciences Division, Newcastle University, Newcastle-upon-Tyne, UK; 2 Heart Institute, Johns Hopkins All Children's Hospital, St. Petersberg, FL, USA; 3 Department of Paediatric Cardiac Surgery, Birmingham Women's and Children's Hospital, Birmingham, UK

**Keywords:** mitral valve, aortic root, cardiac pathology, functionally univentricular heart, hypoplastic left heart complex

## Abstract

The recent special issue of the *World Journal for Pediatric and Congenital Heart Surgery* devoted to hypoplastic left heart syndrome, and its related anomalies, contained significant information of great clinical relevance. Very little attention, however, was devoted to the integrity of ventricular septum as providing a criterion to distinguish between the phenotypes to be included within the syndrome, as opposed to the related anomalies. In this commentary, we summarize the evidence in support of the notion that the phenotypes to be included within the syndrome can be interpreted on the basis of an acquired disease of fetal life. We suggest that it is the integrity of the ventricular septum that provided the major criterion for the distinction between the lesions making up the syndrome and the related anomalies. The subsets of lesions to be included within the syndrome can then be recognized in terms of the time, subsequent to the closure of the embryonic interventricular communication, at which the left ventricle ceased its growth relative to the remainder of the cardiac components. On this basis, it is possible to recognize the combinations of aortic and mitral atresia, mitral stenosis with aortic atresia, combined mitral and aortic stenosis, and hypoplasia of the left ventricle with commensurate hypoplasia of the aortic and mitral valves; the latter combination now recognized as the hypoplastic left heart complex.

## Commentary

The *World Journal for Pediatric and Congenital Heart Surgery* has recently published a special issue devoted to the proceedings of a symposium organized by the World University for Pediatric and Congenital Heart Surgery on hypoplastic left heart syndrome and its related anomalies. There are many important issues addressed within the multiple reviews that make up the issue. One topic that surely requires more attention, however, is ventricular septal integrity. In our opinion, this feature is central to the differentiation between the lesions deemed to belong within the syndrome as opposed to the “related anomalies.” As Marshall Jacobs indicates in his review of the early history of the overall group of lesions,^1^ the term “Hypoplastic Left Heart Syndrome” was first used by Noonan and Nadas in their investigation published in 1958.^2^ As also indicated by Jacobs, Maurice Lev had earlier grouped together the lesions discussed by Noonan and Nadas under the banner of “hypoplasia of the aortic tract complexes.” In his initial study, Lev had included cases with aortic hypoplasia in the setting of deficient ventricular septation. When revisiting the topic of left ventricular hypoplasia in 1966,^3^ he distinguished between those hearts with intact as opposed to deficient ventricular septums. The significance of ventricular septal integrity became clear to us as we reviewed specimens diagnosed and collected by pathologists as being part of the syndrome.^4,5^ Within the special issue, Tchervenkov and his colleagues^6^ identify inconsistent use of terminology as one of the controversies and obstacles underscoring the understanding of the topic, highlighting, in particular, the “incomplete definition of the cardiac phenotypes of hypoplastic left heart syndrome.” We are in total support of this statement. In this regard, the definition for the syndrome currently provided by the International Paediatric and Congenital Cardiac Code rightly emphasizes the requirement for the presence of essentially concordant atrioventricular and ventriculo-arterial connections. It does not, however, include an intact ventricular septum as one of the criteria.^7^ Tchervenkov and colleagues,^6^ in their review, however, listed multiple phenotypes allegedly belonging to the syndrome when the ventricular septum was deficient. Their suggestions are at odds with our own experiences.^4,5^ In addition, they provided no criteria to indicate when the left ventricle, in the setting of deficient ventricular septation, should be considered sufficiently hypoplastic to justify inclusion within the syndrome.

**Figure 1.**
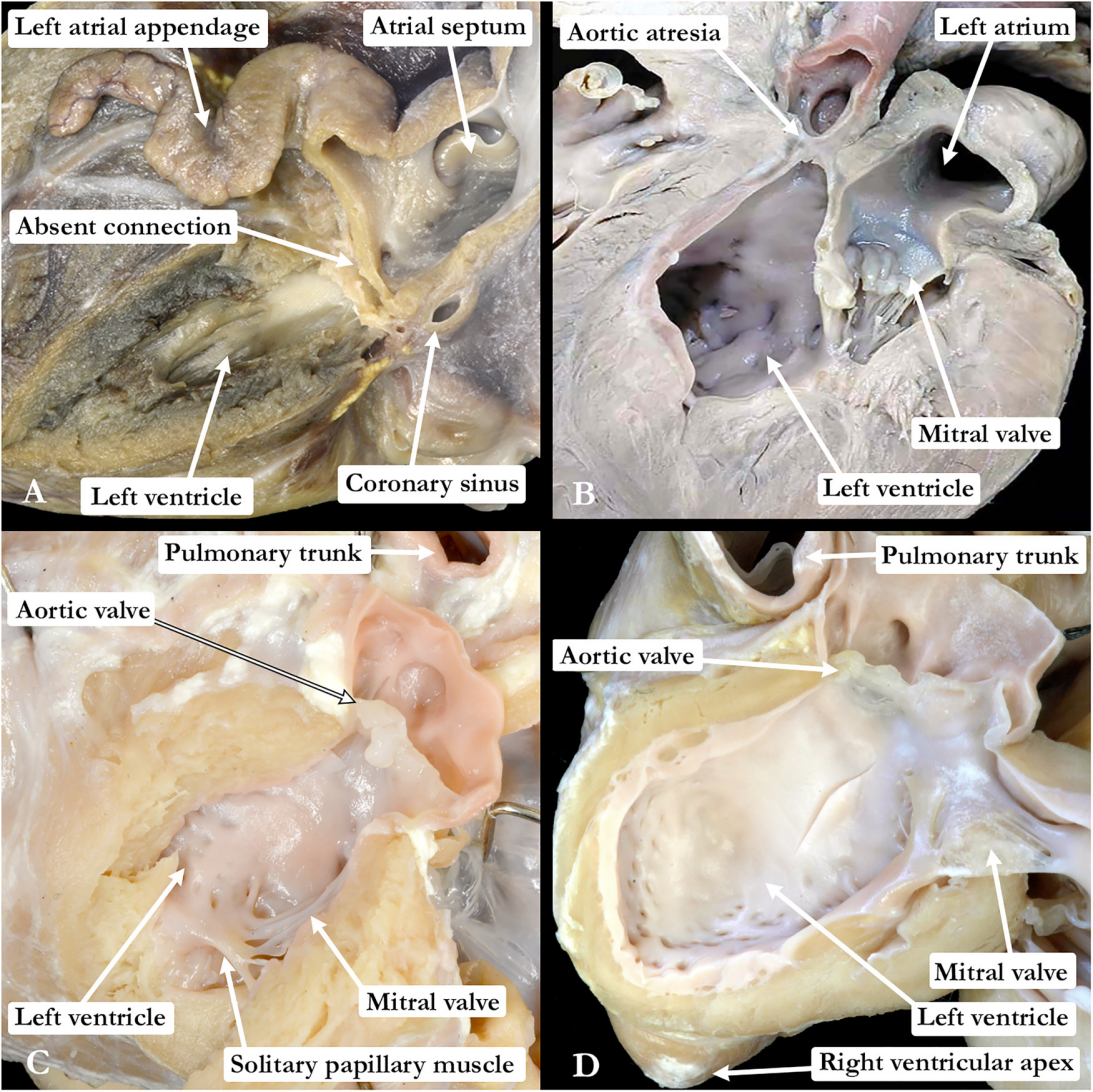
The images in the four panels show the phenotypic features of the variants we would suggest are appropriately grouped to make up the so-called “hypoplastic left heart syndrome.” (A) Combined aortic and mitral atresia, with (B) aortic atresia with mitral stenosis, (C) combined aortic and mitral stenosis, and (D) the features of so-called “hypoplastic left heart complex.” Only in the heart with mitral atresia is the endocardial lining of the left ventricle devoid of fibroelastosis.

Our analyses indicated that an intact ventricular septum was critical in determining the phenotypes to be included within the syndrome, as opposed to those to be listed as related anomalies. The integrity of the septum points to a common thread grouping these lesions, namely a developmental insult that occurs after the completion of ventricular septation. Recognition of this fact permits the hypoplastic left heart syndrome to be interpreted as an acquired disease of fetal life. According to the morphology of the hypoplastic left ventricle, the phenotypes represent a spectrum of malformation (Figure 1). The subgroups are those of combined aortic and mitral atresia (Panel A), aortic atresia with mitral stenosis (Panel B), combined aortic and mitral stenosis (Panel C), and left ventricular hypoplasia, with an intact ventricular septum, but with the sizes of the mitral and aortic valves proportionate to the size of the left ventricle (Panel D). The latter subgroup is now recognized as the so-called hypoplastic left heart complex.^6,8^ Patients with this phenotype are particularly pertinent to the issue of biventricular repair as opposed to univentricular palliation. The findings are equally significant with regard to the morphogenesis and genetic basis of the various lesions. As already indicated, the inference can be made that the severity of the lesions reflects the time, during gestation, when the left ventricle no longer grows in concert with the remainder of the heart. And, again as already stated, we consider it reasonable to presume that whatever underscored the onset of left ventricular hypoplasia, this must have occurred subsequent to the closure of the embryonic interventricular communication. This is not the case when left ventricular hypoplasia is found with a deficient ventricular septum. Nor are the typical gross pathological changes characteristic of the syndrome in the setting of mitral stenosis, notably the presence of the fibroelastotic lining, found when the left ventricle is hypoplastic in the presence of a ventricular septal defect.

It is our opinion, therefore, that the evidence available from pathological archives dictates that an intact ventricular septum should now be added to the definition provided for both hypoplastic left heart syndrome and hypoplastic left heart complex. As was stated by Howell and colleagues in their contribution to the special issue,^9^ “it was Maurice Lev in 1966 who noted that the presence of the VSD created a different clinical entity to that of aortic atresia associated with HLHS.” We would argue that the same argument holds sway for all the lesions that might now appropriately be grouped as belonging to the syndrome when the ventricular septum is intact. The integrity, or otherwise, of the ventricular septum impacts on the hemodynamics of any given feature, with further implications relating to ventricular interactions, development, genetics, the fate of the pulmonary vasculature, protocols for treatment, collection of data, and outcomes. For all of these reasons, we consider it to be preferable to exclude individuals with a physiologically significant ventricular septal defect as belonging to the syndrome, while recognizing, of course, that such individual can exhibit significant left ventricular hypoplasia. As is emphasized throughout the special issue, such patients can have similar clinical presentation, evaluation, and management as those we suggest can now be included within the syndrome. If we are to identify the different genes that underscore the onset of hypoplasia of the left ventricles, nonetheless, it will be essential to differentiate between those individuals with or without a ventricular septal defect. If a “blanket term” is required for the overall group, then it should simply be left ventricular hypoplasia, with the syndrome restricted to those having such hypoplasia in the setting of an intact ventricular septum. The currently accepted definition for the syndrome excludes individuals with deficient atrioventricular septation. If this is the case, is it not equally appropriate to exclude those with hemodynamically significant deficient ventricular septation?
